# DOCK2 as a novel CD11c ligand in neutrophils to regulate reactive oxygen species production

**DOI:** 10.3389/fimmu.2025.1692451

**Published:** 2025-11-05

**Authors:** Sophia Koutsogiannaki, Lifei Hou, Fahd Alhamdan, Mitra Mastali, Christopher Murray, Jennifer Van Eyk, Kazufumi Kunimura, Koichi Yuki

**Affiliations:** ^1^ Department of Anesthesiology, Critical Care and Pain Medicine, Boston Children’s Hospital, Boston, MA, United States; ^2^ Department of Anaesthesia and Immunology, Harvard Medical School, Boston, MA, United States; ^3^ Smidt Heart Institute, Advanced Clinical Biosystems Research Institute, Cedars-Sinai Medical Center, Los Angeles, CA, United States; ^4^ Department of Immunobiology and Neuroscience, Division of Immunogenetics, Medical Institute of Bioregulation, Kyushu University, Fukuoka, Japan

**Keywords:** integrin, CD11c, DOCK2, neutrophil, reactive oxygen species (ROS)

## Abstract

CD11c (integrin αX) is one of the β_2_ integrin members traditionally recognized as a dendritic cell marker. It forms the CD11c/CD18 heterodimer—also known as complement receptor 4 (CR4)—and mediates ligand binding to complement fragments, fibrinogen, and intercellular adhesion molecules *in vitro*. Although its expression on dendritic cells and a subset of macrophage populations has been well recognized historically, recent findings reveal that it demonstrates a broader expression profile, including in neutrophils. In neutrophils, CD11c is predominantly intracellular, suggesting a non-canonical role beyond cellular adhesion. We previously identified IQGAP1 as an intracellular binding partner of CD11c/CD18, implicating this interaction in neutrophil maturation. Here, mature CD11c-deficient neutrophils displayed impaired reactive oxygen species (ROS) generation while maintaining normal phagocytosis, indicating a selective defect in oxidative burst. Given the central role of NADPH oxidase and Rac activation in ROS production, we hypothesized that CD11c would influence this pathway. Phosphoproteomic profiling revealed reduced phosphorylation of the Rac guanine nucleotide exchange factor DOCK2 in CD11c-deficient neutrophils upon phorbol 12-myristate 13-acetate (PMA) stimulation. The analysis involving immunoprecipitation and proteomics confirmed a CD11c–DOCK2 association. These results supported a model in which CD11c would directly engage DOCK2 to promote Rac activation and NADPH oxidase function, uncovering a novel integrin-mediated mechanism regulating neutrophil effector activity. This work expands the functional repertoire of CD11c and provides a new insight into integrin signaling in innate immunity.

## Introduction

CD11c, also known as integrin αX, is a well-characterized adhesion molecule that has been extensively studied as a classical surface marker for dendritic cells in the field of immunology (1). CD11c heterodimerizes with the β_2_ integrin subunit CD18 to form the CD11c/CD18 complex, also referred to as complement receptor 4 (CR4) (2, 3). This integrin complex undergoes conformational changes that allow it to bind to a variety of ligands *in vitro*, including complement fragments, fibrinogen, and intercellular adhesion molecules (4–9). While CD11c expression has been traditionally appreciated on dendritic cells and certain macrophage populations, emerging evidence indicates that its expression is much broader than previously appreciated, extending to other leukocyte subsets under specific physiological or pathological conditions (10–14).

Our recent work and others’ have expanded the understanding of CD11c biology by demonstrating its presence in neutrophils, one of the most abundant and rapidly mobilized effector cells in innate immunity (10). Notably, we found that CD11c expression in neutrophils is predominantly localized to the intracellular compartment rather than the cell surface. We have reported that intracellular CD11c participates in the regulation of neutrophil maturation, a tightly orchestrated process that occurs in the bone marrow and is critical for generating fully functional effector cells. This observation suggested a non-canonical role of CD11c in neutrophil biology beyond the traditional adhesion function as an integrin via its expression on the cell surface. In this context, we identified IQ motif-containing GTPase-activating protein 1 (IQGAP1) as an intracellular ligand of CD11c/CD18 (10). IQGAP1 is a multifunctional scaffolding protein that integrates signals from the cytoskeleton, small GTPases, and adhesion complexes (15), and our findings indicate that the CD11c–IQGAP1 interaction contributes to the maturation program of neutrophils.

Neutrophil maturation is a prerequisite for the acquisition of the diverse effector mechanisms that underpin their role in host defense (16). Mature neutrophils exhibit potent antimicrobial capabilities, including the ability to migrate to infection sites, engulf pathogens through phagocytosis, and destroy them via degranulation and the generation of reactive oxygen species (ROS) (17). In the present study, we observed that mature CD11c-deficient (CD11c KO) neutrophils display a selective defect in ROS generation while retaining normal phagocytic capacity, suggesting that CD11c specifically regulates oxidative burst through distinct signaling pathways even after neutrophil maturation. ROS production, in particular, represents a fundamental antimicrobial mechanism and is mediated by the NADPH oxidase complex (18). Activation of NADPH oxidase involves the assembly of cytosolic and membrane-bound components, a process tightly regulated by signaling cascades that include the activation of small GTPases such as Rac (19). Rac activation promotes electron transfer from NADPH to molecular oxygen, producing superoxide anion and subsequent ROS intermediates (20). Dysregulation of ROS generation can compromise host defense and is implicated in a range of inflammatory and immune-mediated diseases (21). However, the molecular basis by which CD11c contributes to ROS formation remains poorly understood. Given that CD11c is an integrin with established roles in intracellular signaling and that it interacts with scaffold proteins such as IQGAP1 known to modulate cytoskeletal dynamics and GTPase activity (10), it is plausible that CD11c could influence the activation of Rac and, consequently, the NADPH oxidase complex. Yet, the precise signaling pathway connecting CD11c to ROS production has not been fully elucidated. Understanding this connection is important not only for defining the non-canonical functions of CD11c in neutrophils but also for uncovering novel regulatory mechanisms of innate immune effector functions.

To explore this mechanism and to gain further mechanistic insights, we performed phosphoproteomic analysis of wild-type (WT) and CD11c KO neutrophils at baseline and after phorbol 12-myristate 13-acetate (PMA) stimulation. Among PMA-responsive proteins, phosphorylation of Dedicator of Cytokinesis 2 (DOCK2)—a Rac guanine nucleotide exchange factor essential for NADPH oxidase activation (22, 23)—was markedly reduced in CD11c KO neutrophils. STRING analysis predicted a direct CD11c–DOCK2 interaction, which we confirmed by immunoprecipitation and mass spectrometry (MS). These findings led us to hypothesize that CD11c would regulate ROS production through direct interaction with DOCK2, thereby promoting Rac activation and NADPH oxidase function. The present study aims to define the CD11c–DOCK2 interaction and its role in neutrophil oxidative burst, providing a new insight into integrin-mediated control of innate immune effector mechanisms.

## Methods

### Mice

C57BL/6J mice (wild type) were purchased from the Jackson Laboratory. CD11c KO mice were kindly given by Dr. Christie Ballantyne (Baylor University). DOCK2 KO mice were previously described (24). They were housed under specific pathogen-free conditions, with 12-h light and dark cycles. All the animal protocols were approved by the Institutional Animal Care and Use Committee (IACUC) at Boston Children’s Hospital.

### Bone marrow neutrophil isolation

Bone marrow (BM) cells were flushed from the femurs. After red blood cells were lysed, BM cells were fractionated by Percoll gradient (63% and 85%) centrifugation (400×*g*, 30 min). The interface was collected and washed, representing total neutrophils with a purity higher than 90%. To compare protein expression, ROS generation, and phagocytosis between the genotypes, immature and mature neutrophils from BM cells were also sorted using the FACSAria system (BD Biosciences- Franklin Lakes, USA) and verified by Giemsa staining. We identified pre-neutrophils as Lin^−^Gr-1^+^CD11b^+^CXCR4^+^c-kit^int^CXCR2^−^, immature neutrophils as Lin^−^Gr-1^+^CD11b^+^CXCR4^−/low^c-kit^low/−^CXCR2^−^, and mature neutrophils as Lin^−^Gr-1^+^CD11b^+^CXCR4^−/low^c-kit^low/−^CXCR2^+^ as previously done (10, 25, 26).

### Reactive oxygen species formation

Mouse mature neutrophils (2 × 10^5^ in 200 µL) were cultured in complete RPMI 1640 at 37°C for 30 min. Dihydrorhodamine-123 (1 µM; Sigma-Aldrich-St. Loise, USA) was added for 5 min at 37°C. Neutrophils were washed once. PMA (100 nM) was added, and the cells were incubated for an additional 30–60 min at 37°C. After one wash, the cells were resuspended in cold PBS with 1% FCS for the detection of ROS-induced rhodamine-123 (DHR123) on a FACSCanto system (BD Biosciences).

### Phagocytosis

The neutrophil phagocytosis was done using the Phagotest Kit (Glycotope Biotechnology, Heidelberg, Germany). Mouse mature neutrophils were cultured in complete RPMI 1640 on ice for 10 min, followed by the addition of FITC-*E. coli*. Then, the neutrophil suspension was kept on ice as a cold control or was incubated at 37 °C using a water bath for 30 min. At the end of the incubation, cells were transferred back to ice, quenched, and washed. Cells were suspended in PBS/1% PFA, measured by FACSCanto (BD Biosciences), and analyzed by FlowJo software (Tree Star- Ashland, USA).

### Phosphoproteomics

#### Proteomics sample digestion

Samples were lysed in 6 M of urea, 1 M of ammonium bicarbonate, and 5% SDS lysis buffer and sonicated for 10 min at 70% power using a QSonica Q800 sonicator. Samples were cleared by centrifugation at 20,000×*g* for 10 min, and protein concentration was measured by BCA. Samples were digested by an automated SP3 protocol adapted to a Beckman i7 workstation. Bead aliquoting, reduction, alkylation, digestion, and elution were all performed on-deck with a 96-well plate format. Briefly, 50 μg of protein in 40 μL of the previously mentioned lysis buffer was reduced with the addition of 10 μL of 200 mM dithiothreitol and incubated for 30 min at 37°C with shaking at 300 rpm, then alkylated with 10 μL of 400 mM iodoacetamide at room temperature for 15 min in the dark. The volume was brought to 70 μL with Tris–HCl pH 8, then 5 μL of bead suspension [10:1 mass ratio of beads to protein, 1:1 mixture of hydrophilic/hydrophobic beads (Cytiva- Marlborough, USA)] was aliquoted into the samples using the span-8 pipetting head with constant agitation of the bead reservoir between transfers. Samples were brought to 50% acetonitrile (ACN) and incubated for 18 min, and then the solvent was removed on-magnet. The samples were rinsed with 2× 80% ethanol and then 2× ACN with 200 μL volumes each. After the solvent was completely removed, the samples were resuspended in 50 mM of Tris–HCl pH 8 and 10 mM of CaCl_2_ with trypsin at a 1:20 ratio. The samples were bath-sonicated for 5 min and then incubated for 18 h at 37°C and 1,200 rpm overnight. After digestion, the samples were then removed from the beads and brought up to 80% ACN, 1 M of glycolic acid, and 5% trifluoroacetic acid for phosphoenrichment.

#### Phosphoenrichment

Fifty microliters of MagReSyn^®^ IMAC beads were equilibrated in 80% ACN, 1 M of glycolic acid, and 5% trifluoroacetic acid and added to the peptide mixture. Samples were incubated with shaking at room temperature for 30 min; placed on a magnetic rack; washed with 1× 80% ACN, 1 M of glycolic acid, 5% trifluoroacetic acid, 2× 80% ACN, 1% trifluoroacetic acid, 2× 10% ACN, and 0.2% trifluoroacetic acid; and eluted with 1.25 M of ammonium hydroxide. Samples were then acidified with formic acid for liquid chromatography (LC)-MS/MS analysis.

#### LC-MS/MS analysis

Data-dependent acquisition (DDA) analysis was performed on an Orbitrap Exploris 480 (Themo Scientific-Waltham, USA) mass spectrometer interfaced with an EASY-Spray™ ionization source (Thermo Scientific, ES081) coupled to a Vanquish Neo ultra-high-pressure chromatography system with 0.1% formic acid in water as mobile phase A and 0.1% formic acid in acetonitrile as mobile phase B. Peptides were separated at a constant flow rate of 15 µL/min with a linearly increasing gradient of 4%–28% B for 0–52 min and 28%–40% B from 52 to 55 min and then flushed with 98% B from 55 to 60 min. The column used was a Thermo Scientific™ µPac™ HPLC column with a 200-cm bed length (P/N: COL-NANO200G1B). Source settings were set to 3,500 V with the ion transfer tube temperature set to 275°C. MS1 resolution was set to 120,000 with automatic gain control (AGC) target set to custom and normalized AGC target set to 300%. The radiofrequency (RF) lens was set to 50% with charge states 2–6 included and an intensity threshold filter of 8.0e3. MS2 resolution was set to 30,000 with an isolation window of 2 *m*/*z*, a normalized HCD collision energy of 30%, and a custom AGC target of 80% with a custom maximum injection time mode set to 100 ms. All data were acquired in profile mode using positive polarity.

#### Data analysis

MS raw data files were searched against UniProt mouse reviewed protein sequence entries using the FragPipe Analyst LFQ-phospho pipeline (27). Mass shift of 79 Da on Ser, Thr, and Tyr was used to identify phosphorylated peptides.

### Immunoprecipitation

Mouse bone marrow-derived mature neutrophils were collected and lysed on ice in RIPA buffer (Thermo Fisher Scientific) for 30 min, followed by centrifugation at 15,700×*g* for 5 min at 4°C. The collected lysates were subjected to immunoprecipitation using the Dynabeads Protein A Immunoprecipitation Kit (Invitrogen-Waltham, USA). Briefly, Dynabeads were resuspended on a roller for 5 min, and 50 µL of beads were transferred to a microcentrifuge tube. After magnetic separation and removal of the supernatant, beads were incubated with anti-mouse CD11c monoclonal antibody (Cell Signaling #97585; Biolegend N418) or anti-ELMO1 antibody (Invitrogen) in a 1:50 dilution in 200 µL of antibody binding and washing buffer, for 30 min at room temperature with rotation. Antibody-coated beads were washed once and subsequently incubated with cell lysates with rotation for 1 h at room temperature. Following incubation, supernatants were collected for further analysis, while bead–antibody–antigen complexes were washed three times with 200 µL of washing buffer. Complexes were resuspended in 100 µL of washing buffer, transferred to a clean tube, and eluted in 20 µL of elution buffer plus 10 µL 2× Laemmli sample buffer supplemented with β-mercaptoethanol. Samples were heated at 95°C for 10 min, and eluates were collected by magnetic separation and resolved on 4%–20% Tris-glycine gels (Invitrogen). For Western blotting, proteins were transferred onto a nitrocellulose membrane, blocked with 5% blocking buffer for 1 h at room temperature, and probed with anti-DOCK2 antibody (Santa Cruz Biotechnology-Dallas, USA) at a 1:500 dilution. Membranes were incubated with HRP-conjugated secondary antibodies, washed extensively, and visualized by enhanced chemiluminescence.

### GST-CD11c I domain purification

Recombinant human CD11c I domain containing I333G mutant cloned into pGEX-2T vector was expressed in *Escherichia coli* BL21 (DE3) (28). GST-tagged CD11c I domain I333G was purified with glutathione Sepharose bead column following culturing in bacterial cells (29).

### Mass spectrometry analysis

Proteins associated with activated CD11c I domain were pulled down using glutathione Sepharose bead from HL-60 cell lysates using GST-tagged human CD11c I domain I333G. Proteins resolved on SDS-PAGE were subjected to in-gel tryptic digestion (30). Extracted peptide samples were analyzed by the nano-flow liquid chromatography coupled to an Orbitrap Fusion mass spectrometry (nLC-MS/MS, Thermo Scientific) using a Proxeon Easy nLC 1000 Nano-UPLC system (Thermo Scientific) with a Top-12 DDA as we previously described (10, 30). Raw mass spectra data were analyzed by MaxQuant searching software (version 1.5.3.12) coupled to the integrated Andromeda search engine (31, 32). Peptides and proteins were identified with a statistics-based scoring algorithm and filtered at 1% false discovery rate (FDR) by searching against the UniProt human database (downloaded 19 September 2018) in concatenation with the reversed decoy database. Mass tolerance in full MS spectra was set to ± 4.5 ppm, and mass tolerance in MS/MS spectra was set to ± 0.5 Da. Trypsin was specified as the enzyme with a maximum missed cleavage of 2. Fixed modification includes cysteine carbamidomethylation, and variable modifications include methionine oxidation and protein N-acetylation.

### Statistical analysis

Data were analyzed as indicated in the corresponding figure legends. Statistical significance was defined as *p <*0.05. All the statistical calculations were performed using PRISM 10 software (GraphPad Software, La Jolla, CA, USA).

## Results

### Mature CD11c KO neutrophils had impairment in ROS production, but not phagocytosis

**Figure 1:**
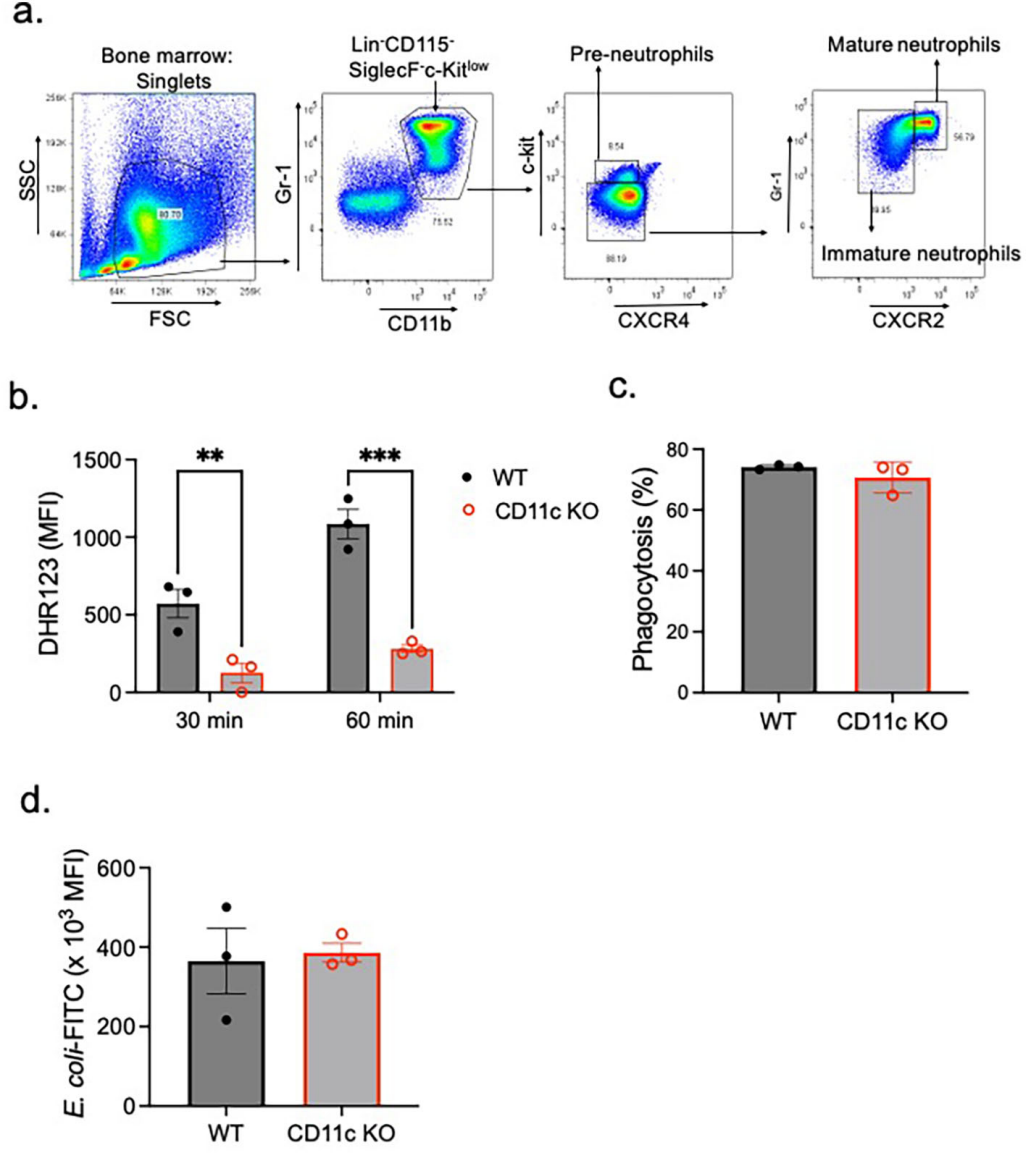
ROS and phagocytosis by mature WT and CD11c KO mature neutrophils. **(A)** Bone marrow mature neutrophils were sorted as Lin^−^Gr-1^+^CD11b^+^CXCR4^−/low^c-kit^low/−^CXCR2^+^ population (25, 26). Using mature neutrophils, **(B)** ROS formation and **(C, D)** phagocytosis were tested. **(B)** ROS formation was tested 30 and 60 min after PMA stimulation. Difference in DHR123 mean fluorescence intensity (MFI) between the PMA stimulation condition and control is shown. **(C, D)** Regarding phagocytosis, we presented % of mature neutrophils that phagocytized *E. coli*-FITC **(C)** and the MFI of phagocytized *E. coli*-FITC **(D)**. Data were shown as mean ± S.E. Each dot represents the average value of each mouse. **(B)** Two-way ANOVA with Bonferroni *post hoc* analysis was used for statistical analysis. **p < 0.01, ***p < 0.001. **(C, D)** The Student’s *t*-test was used for statistical analysis. No significance was observed.

As CD11c affects neutrophil maturation, we sorted mature neutrophils from the BM of WT and CD11c KO mice (**Figure 1A**). While ROS production was attenuated in CD11c KO mature neutrophils (**Figure 1B**), phagocytosis was comparable between WT and CD11c KO neutrophils (**Figures 1C, D**).

### The phosphorylation of DOCK2 was attenuated in CD11c KO mature neutrophils

**Figure 2:**
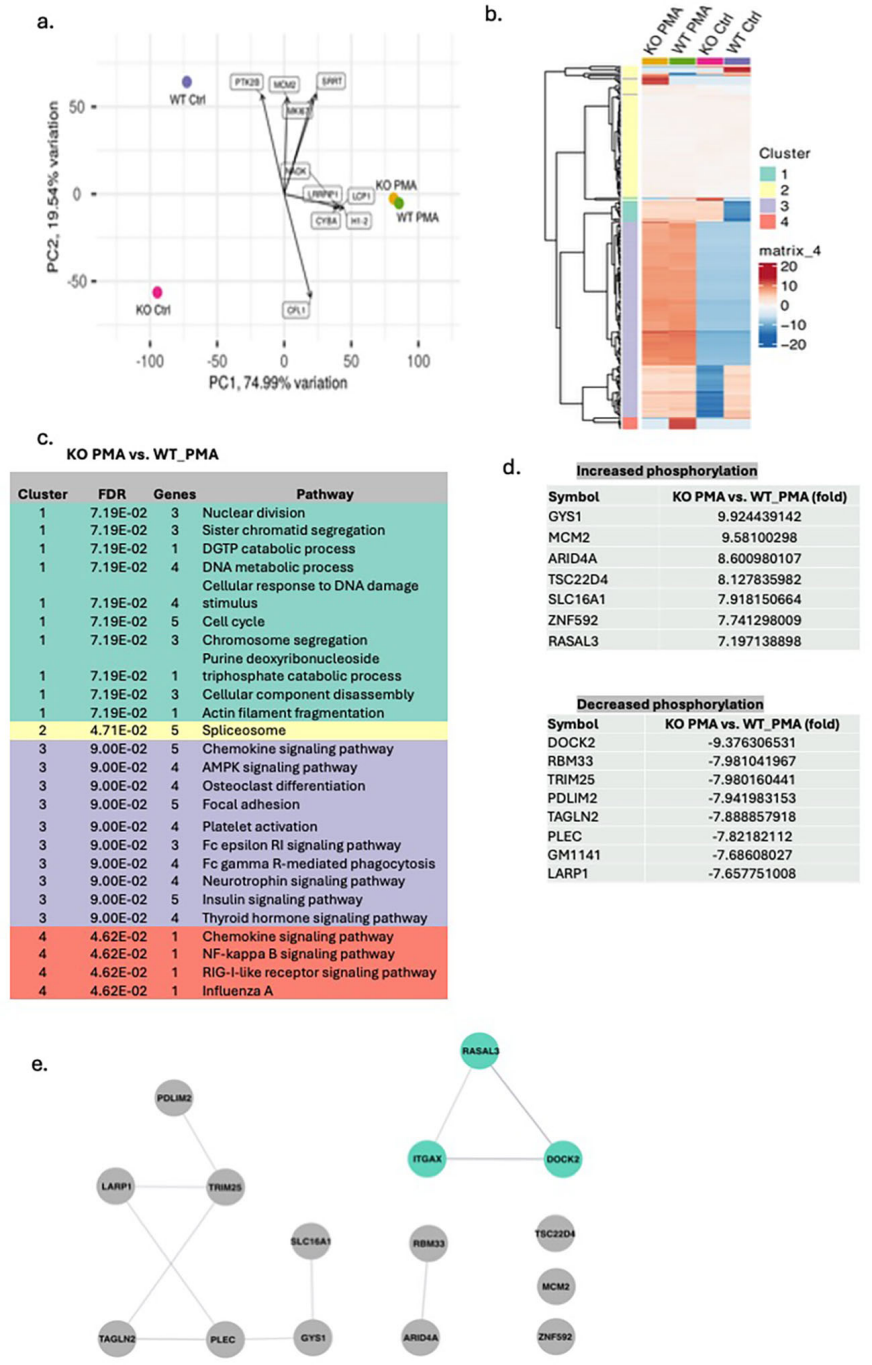
Phosphoproteomics of WT and CD11c KO mature neutrophils stimulated by PMA. The data from the phosphoproteomics experiment were subjected to **(A)** principal component analysis (PCA) and **(B)** cluster analysis. **(C)** In each cluster, functional annotation was also performed comparing CD11c mature neutrophils stimulated by PMA versus WT mature neutrophils stimulated by PMA. **(D)** The list of proteins whose difference in phosphorylation between WT and CD11c KO neutrophils was noted only in PMA stimulation conditions. **(E)** STRING analysis of the proteins in **(D)**. Itgax = CD11c.

PMA stimulation is known to trigger a cascade of protein phosphorylation events (33–35). To investigate this, we performed phosphoproteomic analysis following PMA treatment. At baseline, phosphorylation profiles differed markedly between mature WT and CD11c KO neutrophils; however, PMA stimulation reduced these differences, resulting in a more similar phosphorylation pattern between the two groups (**Figures 2A–C**). This finding suggests that most phosphorylation differences observed at baseline between WT and CD11c KO neutrophils are unlikely to play a major role in PMA-mediated signaling. We next identified the phosphorylated proteins specifically associated with PMA stimulation (**Figure 2D**). Phosphorylation of DOCK2, RBM33, TRIM25, PDLIM2, TAGLN2, PLEC, GM1141, and LARP1 was markedly reduced in PMA-stimulated CD11c KO neutrophils compared with PMA-stimulated WT neutrophils. To explore whether a subset of these proteins might directly interact with CD11c, we performed STRING analysis, which indicated a potential interaction between CD11c and DOCK2/RASAL3 (**Figure 2E**). As RASAL3 deficiency rather increases ROS production (36), we focused on DOCK2 going forward.

### DOCK2 interacted with CD11c

**Figure 3:**
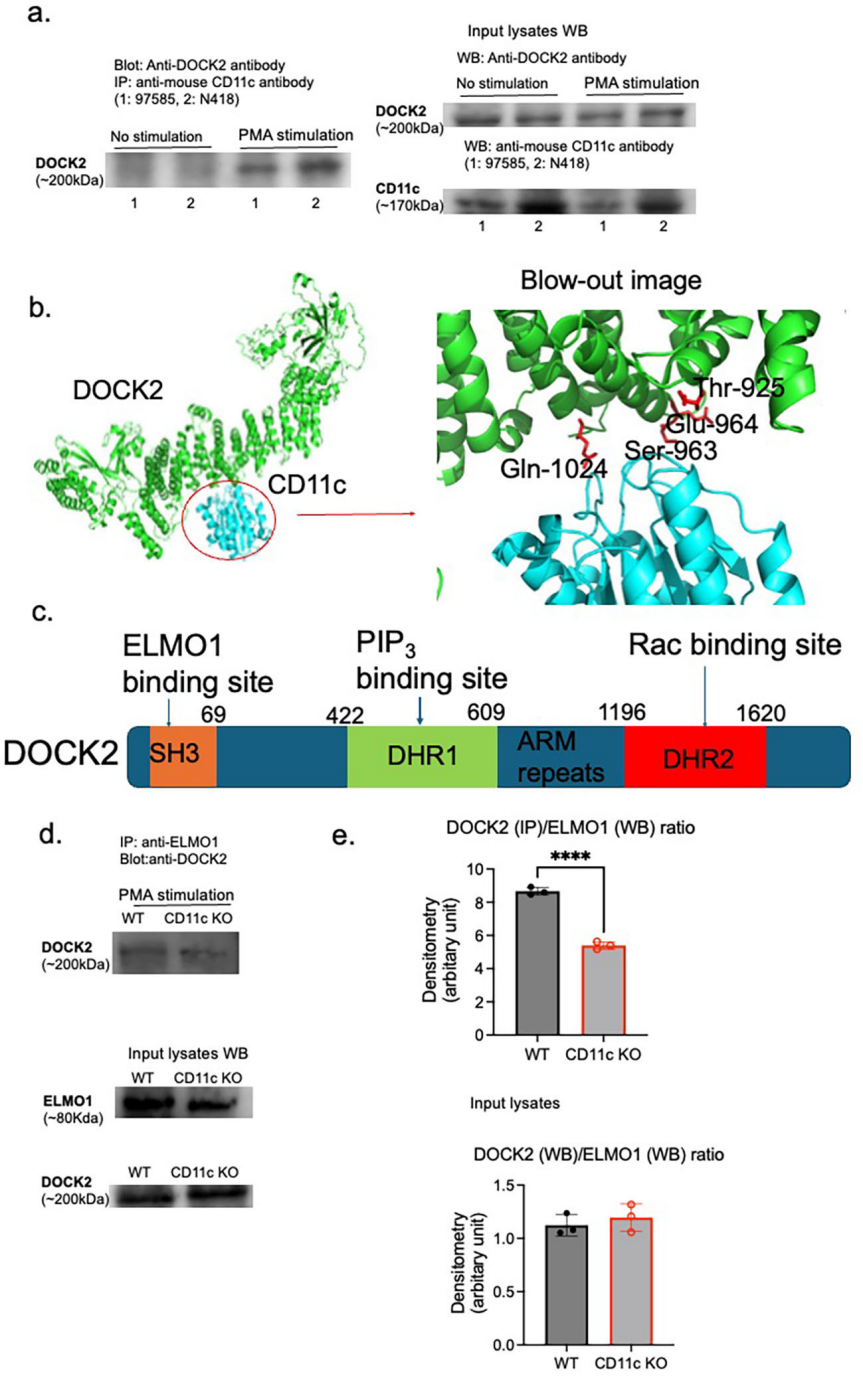
Interaction between CD11c and DOCK2. CD11c and DOCK2 interaction was probed by **(A)** immunoprecipitation and **(B)** *in silico* structure simulation. **(A)** Left panel: Lysed WT mature neutrophils with or without PMA stimulation (100 µg of protein lysates per lane were used for immunoprecipitation input) were immunoprecipitated with two different anti-mouse CD11c antibodies (97585 for 1 and N418 for 2) and probed with anti-DOCK2 antibody. Right panel: The expression of DOCK2 and CD11c in input lysates was shown. **(B)** AlphaFold 2 was used for *in silico* simulation. **(C)** The scheme of DOCK2 domains along with their binding proteins. SH3, N-terminal Src homology 3; ARM, armadillo; DHR, DOCK homology region. It is known that the binding of ELMO1 to DOCK2 at SH3 will enhance Rac activation. **(D, E)** The lysates of WT and CD11c KO mature neutrophils stimulated with PMA (100 µg of protein lysates each) were immunoprecipitated with anti-ELMO1 antibody, followed by probing with anti-DOCK2 antibody. In addition, ELMO1 and DOCK2 expression levels in input lysates were shown. Images were representatives of two independent experiments **(D)**. **(E)** Using densitometry analysis, the ratio of DOCK2/ELMO1 was compared between WT and CD11c KO mature neutrophils. On the top panel, we compared DOCK2 expression on IP/ELMO1 expression on WB between WT and CD11c KO mature neutrophils stimulated with PMA. On the bottom panel, we compared DOCK2 expression on WB/ELMO1 expression on WB between WT and CD11c KO mature neutrophils stimulated with PMA. The Student’s *t*-test was used for statistical analysis. ****p < 0.0001.

To test the hypothesis that CD11c directly binds to DOCK2, we performed an immunoprecipitation experiment. In murine neutrophils, we showed that CD11c was co-immunoprecipitated with DOCK2 upon PMA stimulation (**Figure 3A**). *In silico* structural prediction using AlphaFold 2 (AF2) also supported their direct interaction (**Figures 3B**). The structural model suggested that CD11c interacts with DOCK2 at its armadillo (ARM) repeat domain (**Figures 3B, C**). To further validate this interaction, we performed proteomic analysis of proteins immunoprecipitated with the CD11c protein only under PMA stimulation. DOCK2 was among the proteins identified of the proteins identified (data not shown), providing additional evidence for a direct association between CD11c and DOCK2. DOCK2 is a multidomain protein capable of binding multiple partners at distinct sites (**Figure 3C**). Notably, one of these partners, ELMO1 (Engulfment and Cell Motility 1), was also detected in the CD11c immunoprecipitate (**Figure 3D**) and proteomics analysis of proteins immunoprecipiated with CD11c protein (data not shown).

### CD11c KO neutrophils impaired the interaction between ELMO1 and DOCK2

The interaction between DOCK2 and ELMO1 promotes DOCK2 phosphorylation and subsequent binding to Rac, a critical step for NADPH oxidase activation and ROS production. Based on this, we hypothesized that loss of CD11c would disrupt the ELMO1–DOCK2 interaction and thereby reduce DOCK2 phosphorylation. Consistent with this hypothesis, immunoprecipitation experiments revealed reduced association between DOCK2 and ELMO1 in CD11c KO mature neutrophils compared with WT controls under PMA stimulation (**Figures 3D, E**), indicating that CD11c contributes, at least in part, to the formation of the ELMO1–DOCK2 complex.

### DOCK2 deficiency was associated with ROS impairment but did not affect neutrophil maturation or phagocytosis

**Figure 4:**
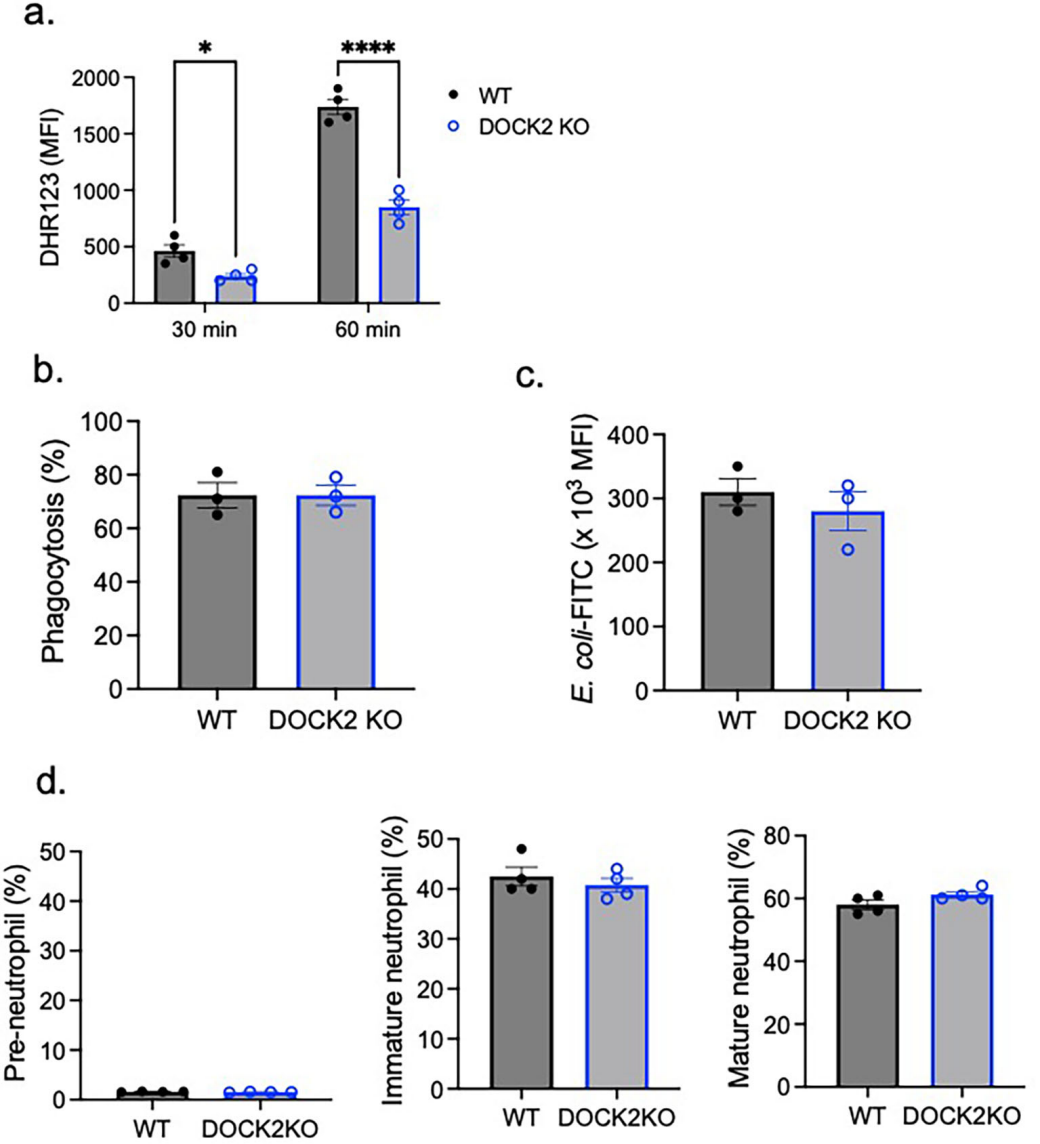
The role of DOCK2 in ROS, phagocytosis, and neutrophil maturation. WT and DOCK2 KO bone marrow neutrophils were tested for **(A)** ROS formation, **(B, C)** phagocytosis, and **(D)** maturation. Using mature neutrophils, **(A)** ROS formation and **(B, C)** phagocytosis were tested. **(A)** ROS formation was tested 30 and 60 min after PMA stimulation. Difference of DHR123 MFI between PMA stimulation condition and the control was shown. **(B, C)** Regarding phagocytosis, we presented % of mature neutrophils that phagocytized *E. coli*-FITC **(B)** and the MFI of phagocytized *E. coli*-FITC **(C)**. **(D)** Pre-neutrophils, immature neutrophils, and mature neutrophils were gated as shown in **Figure 1A**. Pre-neutrophils, immature neutrophils, and mature neutrophils % among the total neutrophils were shown. Data were shown as mean ± S.E. Each dot represents the average value of each mouse. Two-way ANOVA with Bonferroni *post hoc* analysis **(A)** or the Student’s *t*-test **(B–D)** was used for statistical analysis. *p < 0.05, ****p < 0.0001. No statistical significance was observed.

To further validate the role of DOCK2 in CD11c-mediated ROS production, we tested BM neutrophils from DOCK2 KO mice. In line with mature CD11c KO neutrophils, DOCK2 KO neutrophils showed an impairment in ROS production (**Figure 4A**), but not in phagocytosis (**Figures 4B, C**). In addition, we showed that DOCK2 did not affect neutrophil maturation, since no significant difference was observed in the percentage of mature neutrophils between WT and DOCK2 KO mice (**Figure 4D**).

## Discussion

ROS formation is one of the central antimicrobial effector functions of neutrophils, enabling the rapid eradication of invading pathogens. The NADPH oxidase complex, responsible for ROS production, is tightly regulated at multiple levels, including membrane assembly, cytoskeletal rearrangements, and small GTPase activation (17, 18). Among these regulators, Rac activation plays a pivotal role in initiating electron transfer from NADPH to oxygen molecules, resulting in the generation of superoxide anion and subsequent ROS intermediates (19). Our present study identifies CD11c as a critical modulator of ROS generation in mature neutrophils and provides mechanistic evidence linking CD11c to DOCK2.

### CD11c as a regulator of ROS production in mature neutrophils

A previous work from our group and others has established that CD11c is expressed not only in dendritic cells, where it is widely used as their surface marker, but also in neutrophils (37). We previously showed that CD11c influences neutrophil maturation through an interaction with the scaffolding protein IQGAP1, suggesting non−canonical intracellular roles beyond cellular adhesion and migration (10). In this study, we found that CD11c deficiency selectively impairs ROS production in mature neutrophils without affecting phagocytosis. This functional specificity implies that CD11c contributes to ROS generation through distinct intracellular signaling pathways rather than through general disruption of neutrophil effector mechanisms. This finding is consistent with earlier reports showing that integrins can directly regulate NADPH oxidase activity. For example, β_2_ integrin engagement has been shown to trigger Rac activation and promote oxidative burst in monocytes and neutrophils (38–40). However, most of the previous studies around β_2_ integrins focused on LFA1 (CD11a/CD18) or Mac−1 (CD11b/CD18) rather than CD11c/CD18. Our results extend the concept to CD11c, revealing that it may also serve as an upstream modulator of ROS by influencing Rac activation.

### Phospho−proteomics implicates the involvement of DOCK2 in CD11c-mediated ROS formation in mature neutrophils

To uncover potential mediators linking CD11c to ROS generation, we employed phospho−proteomics to compare mature WT and CD11c−deficient neutrophils at baseline and after PMA stimulation. While their phosphorylation patterns at baseline differed substantially, PMA stimulation diminished these differences, suggesting that most of the resting phosphorylation differences between WT and CD11c KO mature neutrophils are not directly relevant to PMA−induced oxidative burst. By focusing on PMA−responsive phosphorylation events, we identified DOCK2 as one of the most significantly affected proteins in CD11c KO neutrophils. DOCK2 is a hematopoietic−specific guanine nucleotide exchange factor (GEF) for Rac that plays an essential role in cytoskeletal remodeling, chemotaxis, and NADPH oxidase activation (22). The importance of DOCK2 in neutrophil biology has been well documented: DOCK2−deficient neutrophils exhibit impaired chemotaxis and defective ROS generation (22). Thus, our observation that DOCK2 phosphorylation was attenuated in CD11c−deficient neutrophils provided a compelling mechanistic link between CD11c and oxidative burst regulation.

### CD11c–DOCK2–ELMO1 interaction as a potential regulatory axis

DOCK2 contains two key functional domains: the DHR1 domain, which binds phosphatidylinositol 3,4,5−trisphosphate (PIP_3_) generated by phosphoinositide 3−kinase (PI3K), and the DHR2 domain, which mediates GDP−to−GTP exchange on Rac (41, 42). In the absence of PI3K activity or PIP_3_ production, Rac activation is significantly reduced (42). Additionally, DOCK2 contains an N−terminal Src homology 3 (SH3) domain that binds to ELMO1. This interaction protects DOCK2 from ubiquitin−mediated degradation and enhances Rac activation by stabilizing DOCK2–Rac binding (43, 44).

Interestingly, our mass spectrometry analysis of CD11c I−domain pull−down eluates identified both DOCK2 and ELMO1 among the interacting proteins. This observation, together with the attenuated DOCK2 phosphorylation in CD11c KO neutrophils, suggests that CD11c may stabilize DOCK2 function by facilitating its association with ELMO1. Indeed, our co−immunoprecipitation experiments revealed greater DOCK2–ELMO1 complex formation in WT neutrophils than in CD11c KO neutrophils, supporting this hypothesis. This finding is mechanistically significant for two reasons. First, the DOCK2–ELMO1 interaction is known to inhibit DOCK2 ubiquitination, thereby preventing its degradation and preserving its Rac−activating capacity. Second, by promoting DOCK2–ELMO1 interaction, CD11c could indirectly enhance Rac activation and NADPH oxidase assembly, resulting in more robust ROS production.

### Integrin-mediated signaling and cytoskeletal regulation

The concept that integrins regulate small GTPase activity through recruitment of GEFs and scaffold proteins is well established in other systems. For example, β_2_ integrin ligation has been linked to Vav−1−mediated Rac activation (45). Similarly, β_1_ integrins can activate Rac through interaction with DOCK180, the founding member of the DOCK family (46). Our findings suggest that CD11c/CD18 may employ an analogous strategy in neutrophils, recruiting or stabilizing DOCK2 via protein–protein interactions to regulate Rac activation. The presence of IQGAP1 as another intracellular CD11c−binding partner further supports the notion that CD11c can participate in cytoskeletal and signaling complexes independent of its cellular adhesive functions.

### Functional specificity of CD11c in ROS versus phagocytosis

**Figure 5:**
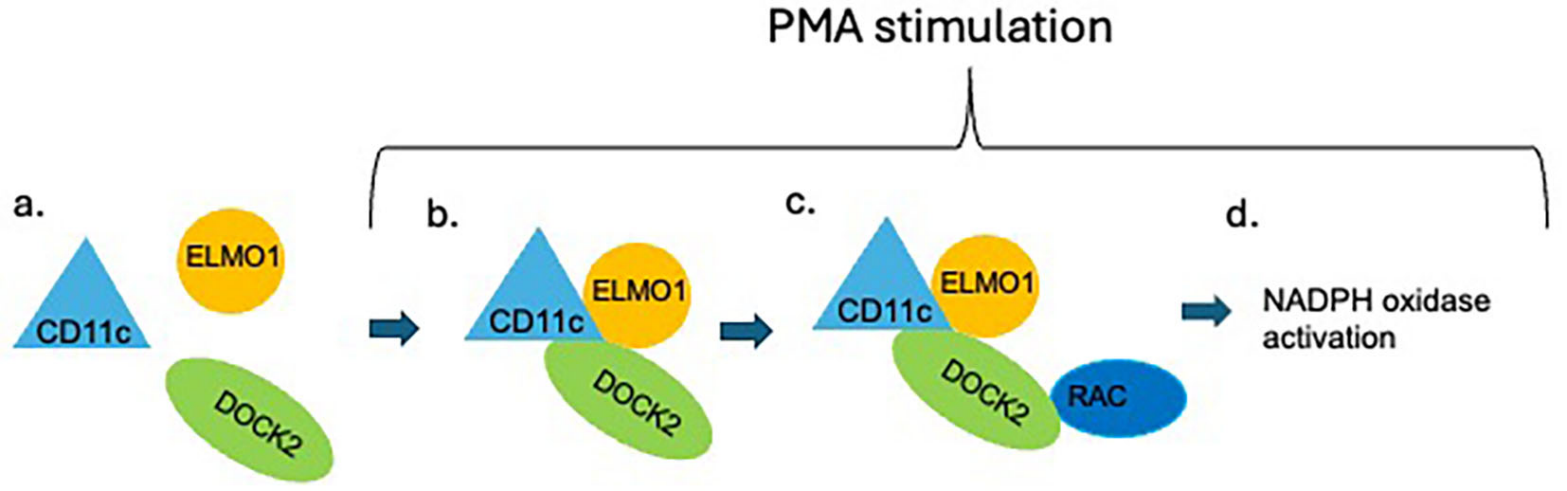
Scheme of the proposed role of CD11c in neutrophil ROS formation. In resting condition, DOCK2 is not activated **(A)**. Upon PMA stimulation, intracellular CD11c facilitates the interaction between ELMO1 and DOCK2 **(B)**, which is known to enhance the interaction between DOCK2 and Rac **(C)**. The DOCK2-Rac interaction activates Rac, which induces NADPH oxidase activation **(D)**.

One striking aspect of our results is that CD11c deficiency selectively impairs ROS generation without affecting phagocytosis. This functional dissociation underscores that CD11c is not a general regulator of neutrophil activation but rather modulates specific signaling pathways. In the context of phagocytosis, other integrins such as Mac−1 may play a dominant role, whereas ROS generation—particularly in response to PMA—may depend more heavily on CD11c−mediated recruitment or stabilization of DOCK2. This specificity may also reflect compartmentalization of signaling complexes, with CD11c preferentially localized to regions of the cell where DOCK2–ELMO1 interactions are initiated, which activates Rac for ROS formation (**Figure 5**).

### Physiological and pathological implications

The identification of a CD11c–DOCK2–ELMO1 axis in neutrophils has important implications for innate immunity. ROS production is essential for bacterial killing, and defects in NADPH oxidase components cause chronic granulomatous disease, characterized by recurrent infections (47). While CD11c deficiency has not been described in human immunodeficiency syndromes, altered CD11c expression or function could theoretically contribute to impaired host defense. Moreover, excessive ROS production has been implicated in inflammatory tissue damage, suggesting that modulation of the CD11c–DOCK2 pathway could represent a therapeutic strategy to fine−tune neutrophil oxidative responses in diseases such as acute respiratory distress syndrome, autoimmune vasculitis, or sepsis. It is well known that ROS can be induced by various stimulants including N-formyl-L-methionyl-L-leucyl-phenylalanine (fMLP), adenosine triphosphate (ATP), and bacterial phagocytosis in addition to PMA (48–50).

### Future directions

While our data support a model in which CD11c promotes ROS production by stabilizing DOCK2–ELMO1 complexes in the murine system, several questions remain. The precise structural interface between CD11c and DOCK2/ELMO1 is not yet defined. Whether this interaction requires CD11c ligand engagement or integrin activation remains to be determined. Additionally, it will be important to investigate whether CD11c influences DOCK2 subcellular localization, as spatial targeting of DOCK2 to PIP_3_−rich membranes is essential for its activity. Exploring whether this pathway contributes to neutrophil function *in vivo* during infection or inflammation will help establish its physiological relevance. Lastly, we did not test in the human system, as currently, CD11c small molecule antagonists are not available.

### Conclusions

In summary, we have identified CD11c as a novel regulator of DOCK2−dependent ROS production in neutrophils. Our results support a model in which CD11c promotes DOCK2 phosphorylation, which subsequently facilitates DOCK2–ELMO1 interaction, and thereby enhances Rac activation and NADPH oxidase assembly. This work expands the functional repertoire of CD11c beyond its classical role in adhesion, positioning it as an important signaling integrin in neutrophil antimicrobial function. Understanding how integrins coordinate with small GTPase regulators to control effector functions may provide new opportunities for modulating immune responses in infection and inflammation.

## Data Availability

The mass spectrometry proteomics data in the study have been deposited to the ProteomeXchange Consortium (https://proteomecentral/proteomexchage.org) via the iProX partner repository with the dataset identifier PXD069967.
